# Interfacial Characterization of Low-Temperature Cu-to-Cu Direct Bonding with Chemical Mechanical Planarized Nanotwinned Cu Films

**DOI:** 10.3390/ma15030937

**Published:** 2022-01-26

**Authors:** Po-Fan Lin, Dinh-Phuc Tran, Hung-Che Liu, Yi-Yi Li, Chih Chen

**Affiliations:** 1 Department of Materials Science and Engineering, National Yang Ming Chiao Tung University, Hsinchu 30010, Taiwan; ivanlin0310@gmail.com (P.-F.L.); trandinhphuc1508@gmail.com (D.-P.T.); c0936390380@gmail.com (H.-C.L.); k8815096@ms52.hinet.net (Y.-Y.L.); 2 Department of Materials Science and Engineering, National Chiao Tung University, Hsinchu 30010, Taiwan

**Keywords:** Cu-to-Cu bonding, chemical mechanical planarization, nanotwinned copper

## Abstract

Copper-to-copper (Cu-to-Cu) direct bonding is a promising approach to replace traditional solder joints in three-dimensional integrated circuits (3D ICs) packaging. It has been commonly conducted at a temperature over 300 °C, which is detrimental to integrated electronic devices. In this study, highly (111)-oriented nanotwinned (nt) Cu films were fabricated and polished using chemical mechanical planarization (CMP) and electropolishing. We successfully bonded and remained columnar nt-Cu microstructure at a low temperature of 150 °C thanks to the rapid diffusion of Cu on (111) surface. We employed a new microstructural method to characterize quantitatively the interfacial bonding quality using cross-sectional and plan-view microstructural analyses. We discovered that CMP nt-Cu bonding quality was greater than that of electropolished nt-Cu ones. The CMP nt-Cu films possessed extremely low surface roughness and were virtually free of pre-existing interface voids. Thus, the bonding time of such CMP nt-Cu films could be significantly shortened to 10 min. We expect that these findings may offer a pathway to reduce the thermal budget and manufacturing cost of the current 3D ICs packaging technology.

## 1. Introduction

Nowadays, three-dimensional (3D) integrated circuits (ICs) technology has been widely employed to enhance the performance of microelectronic devices. A 3D ICs device typically consists of various solder joints. However, such traditional solder joints intrinsically present many reliability issues related to electromigration (EM), the formation of brittle intermetallic compounds (IMCs), circuit shortage, and/or thermomigration [1,2,3,4,5,6,7]. To overcome these challenges, Cu-to-Cu direct bonding has been applied to replace those solder joints [8,9]. Note that Cu-to-Cu bonding with excellent mechanical and conductive properties was typically achieved at temperatures above 300 °C [9,10,11]. However, such bonding temperatures are significantly high and detrimental to microelectronic devices during integration. Thus, investigations on low-temperature Cu-to-Cu bonding are urgently needed. Previously, Liu et al. reported that the bonding temperature could be minimized to 150 °C using nanotwinned Cu (nt-Cu) films [12]. These films could be fabricated using electroplating [12,13,14,15,16] or magnetron sputtering [17,18,19,20,21,22]. Such highly (111)-oriented nt-Cu films possessed the highest diffusivity among the others [12,23]. This breakthrough was expected to reduce the thermal budget and cost of high vacuum manufacturing processes [12,23].

During the operation or fabrication process of electronic devices, Cu joints might be subjected to thermal stress or annealing [24,25]. The progressive evolution of interfacial voids in those joints has been identified [25]. Most of the previous studies qualitatively examined interfacial bonding using cross-sectional imaging [8,11,12,23,26,27,28]. Those studies did not provide detailed information on interfacial voids in terms of shape, size, location, and distribution. To date, a quantitative investigation on the interfacial bonding quality has not been reported. In this study, we used a new microstructural method to quantitatively characterize the interfacial quality of the low-temperature Cu-to-Cu direct bonding. Additionally, it has been reported that the bonding strength and temperature are strongly related to Cu surface roughness [26,29,30]. The reduced surface roughness of Cu films may result in lower bonding temperature and time. Therefore, we employed chemical mechanical planarization (CMP) and electropolishing methods to reduce the surface roughness of the nt-Cu films. We conducted the Cu-to-Cu direct bonding under various conditions (temperature and time). We found that the surface roughness of nt-Cu films treated using CMP was much lower compared to that of the electropolished ones. It led to an enhanced quality of nt-Cu bonding. The bonding time was further shortened as a result of the lower surface roughness of the CMP nt-Cu films.

## 2. Materials and Methods

In this study, a Si wafer, containing a 100-nm Ti adhesion layer and a 200-nm Cu seed film, was used as a substrate. We fabricated nt-Cu films on such substrates using direct current (DC) electroplating [12] with a current density of 80 mA/cm^2^. Detailed chemicals of electrolyte and experimental setup were given in our previous study [12]. The thickness of the electroplated nt-Cu films was controlled as 8 μm. The as-deposited films were then polished using CMP and electropolishing. During the CMP process, a favorable slurry was continuously added into a lapper (Logitech PM5). An applied lapping pressure of 1.5 psi and a polishing rate of 0.02 μm/min were set.

After the CMP process, the samples were cut into physical dimensions of 3 mm × 3 mm. It was then ultrasonically cleansed in a solution of acetone for 5 min. Subsequently, the specimens were further cleaned using a dilute hydrochloric (HCl) acid for 30 s, rinsed in deionized (DI) water, and purged with N_2_ gas. Two samples were attached face to face and transferred to a chamber prior to the bonding process [23]. A pressure of 0.78 MPa was applied to the bonding specimens. The bonding process was conducted in a 10^−3^ torr vacuum level. Bonding temperatures were controlled at 150 °C for 60 min and 200 °C for 30 min, 10 min, and 5 min. X-ray diffraction (XRD, Bruker D2 Phaser, Billerica, MA, USA) and electron back-scattered diffraction (EBSD, JEOL JSM-7800F, Tokyo, Japan) were performed to analyze grain size and crystal orientation. Microstructures and bonding quality was characterized by a focused ion beam (FIB, FEI Nova 2000, Hillsboro, OR, USA). Additionally, an atomic force microscope (AFM, Bruker Innova SPM, Billerica, MA, USA) was employed to obtain the random roughness patterns and determine the surface roughness (*R*_q_) of the nt-Cu samples. The bonding interfaces of the nt-Cu films were examined using a transmission electron microscope (TEM).

**Figure 1:**
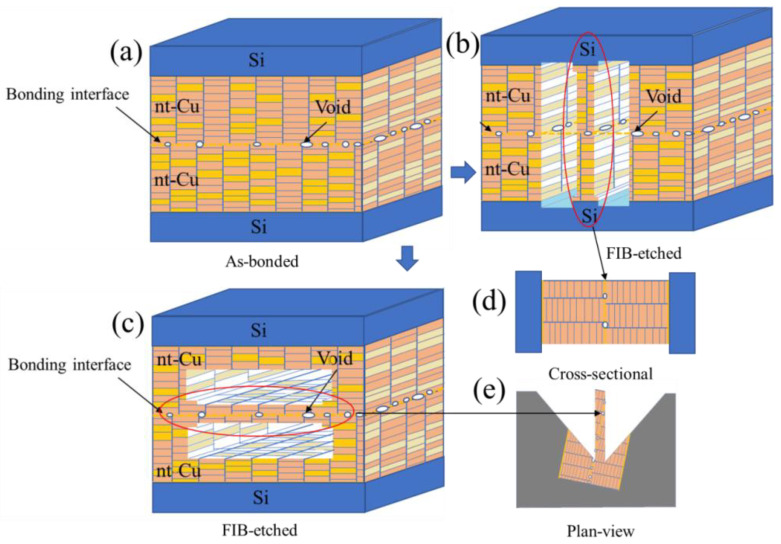
Schematic illustration of the Cu-to-Cu bonding showing various voids at the interface: (**a**) as-bonded; (**b**,**c**) cross-sectional and plan-view FIB-etched; (**d**,**e**) for cross-sectional and plan-view TEM.

**Figure 2:**
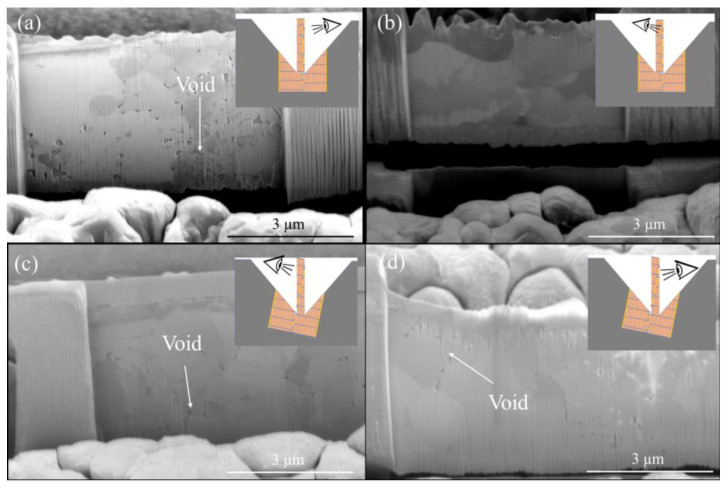
(**a**,**b**) FIB-etched images observed from the back and front of the plan-view TEM sample bonded at 200 °C for 5 min. The inset shows that the bonding interface is parallel with the FIB etching direction. (**c**,**d**) FIB-etched images observed from the back and front of the plan-view TEM sample bonded at 200 °C for 5 min. The inset shows that the bonding interface intersects with the FIB etching direction.

Prior to TEM examination, cross-sectional and plan-view FIB-etched samples were fabricated. Figure 1 shows the schematics of Cu-to-Cu bonding with various interfacial voids. The TEM samples were FIB-etched in two different directions. The former is perpendicular (Figure 1b) and the latter is parallel to the bonding interface (Figure 1c). It is simple to obtain a bonding interface for the cross-sectional sample (Figure 1d) since the etching direction of FIB is always perpendicular to the bonding interface. However, it is of great challenge to prepare a FIB sample, in which the FIB etching direction is parallel throughout the bonding interface (the insets of Figure 2a,b). Note that the size of voids is in a range between 10–100 nm. The thickness of a TEM sample varies from 50 nm to 100 nm. It is complicated to prepare a TEM sample containing a bonding interface with a large area of voids. As shown in the insets of Figure 2c,d, the bonding interface is not parallel to the etching direction. They may intersect with each other. To acquire perfect samples for the TEM examination, the top and bottom of the two FIB-etched sides have some signs of interfacial voids (Figure 2c,d). Using such a technique, we could ensure that the FIB-etched sample contained a large area of the bonding interface. Thus, we were able to characterize quantitatively the void distribution of bonding interfaces using a further TEM.

## 3. Results

### 3.1. Microstructure of nt-Cu Films

**Figure 3:**
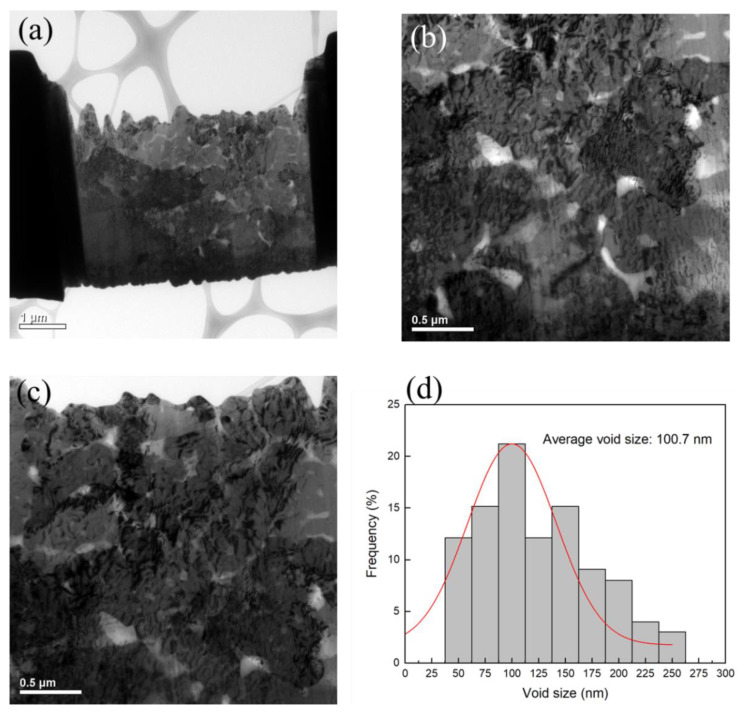
(**a**) Plan-view TEM image of the bonding with (**b**,**c**) enlarged micrographs showing various voids at the bonding interface and (**d**) distribution of interfacial voids.

**Figure 4:**
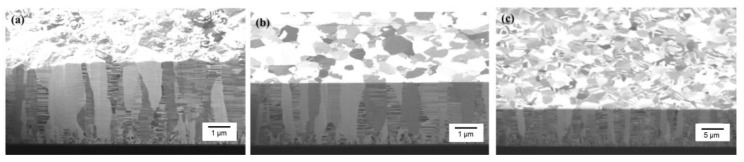
Cross-sectional FIB images of the (**a**) as-electroplated nt-Cu, (**b**) CMP nt-Cu, and (**c**) electropolished nt-Cu films.

**Figure 5:**
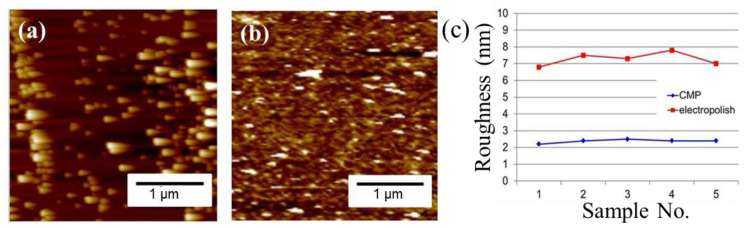
AFM images of the (**a**) as-electropolished and (**b**) CMP nt-Cu films with (**c**) its corresponding surface roughness. The surface roughness (R_q_) is significantly trimmed off from 7 nm to 2.4 nm.

The plan-view TEM images of the Cu-to-Cu films bonded at 200 °C for 30 min are shown in Figure 3a–c. Voids are represented as the brightish spots in the TEM images. It can be seen that various voids were located at the bonding interface and grain boundaries of Cu. The size of voids varies from 30 to 260 nm with an average value of 100.7 nm (Figure 3d). The cross-sectional FIB images of the nt-Cu films with and without planarization are shown in Figure 4. The surface of the as-deposited nt-Cu film exhibited various cone-like shapes (Figure 4a). It was observed that various Cu nanotwins were densely stacked and formed columnar grains. Figure 4b shows a typical microstructure of the nt-Cu films after CMP. For comparison, some samples were electropolished and the typical microstructure is shown in Figure 4c. The nt-Cu film surface was extraordinarily smooth and the fine columnar nt grains remained. Figure 5 shows the AFM micrographs and surface roughness of samples polished using two methods. As shown in Figure 5c, we found that the surface roughness (R_q_ = 2.44 ± 0.12 nm) of samples polished using CMP was extremely lower compared to that of the electropolished samples (R_q_ = 7.33 ± 0.40 nm).

**Figure 6:**
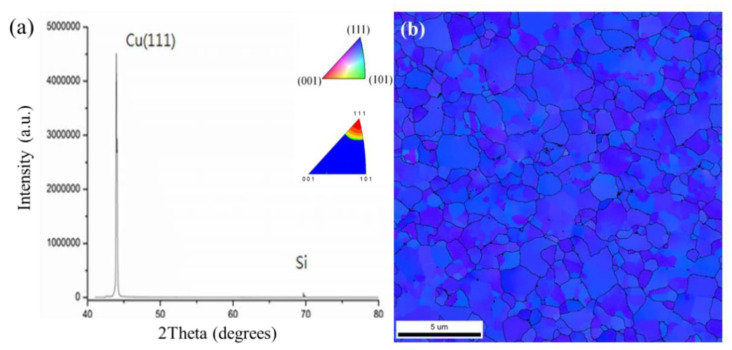
(**a**) XRD pattern and (**b**) plane-view EBSD image of the electroplated nt-Cu films.

The typical grain orientation with an XRD pattern and a plane-view EBSD image of the electrodeposited nt-Cu films are shown in Figure 6. It was found that nt-Cu grains were highly (111)-oriented (Figure 6a). Note that the blue zones in Figure 6b were employed to illustrate the (111)-oriented nt-Cu grains. It is obvious that a highly (111) orientation dominated the nt-Cu surface. An area ratio of 98% of (111)-oriented grains was acquired from the inverse pole figure (IPF) and pole image (Figure 6b). The EBSD observation also supports the above XRD result. Such electroplated nt-Cu films with a high (111)-preferred orientation are expected to exhibit great mechanical performance, excellent thermal stability, and EM resistance [13,14,15,31,32,33,34,35,36,37,38].

### 3.2. Microstructure of Bonding Interface

**Figure 7:**
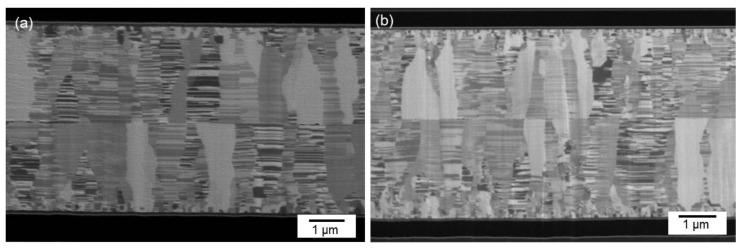
Cross-sectional FIB image of the typical CMP nt-Cu films bonded at (**a**) 150 °C for 60 min and (**b**) 200 °C for 5 min.

**Figure 8:**
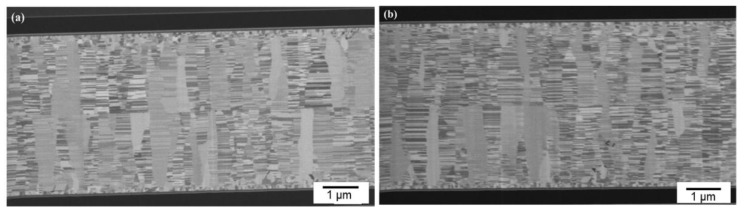
Cross-sectional FIB images (**a**,**b**) of the typical CMP nt-Cu films bonded at 200 °C for 30 min.

Figure 7a presents a typical cross-sectional FIB micrograph of the CMP nt-Cu films bonded at 150 °C for 60 min. We found that the columnar microstructure of nt-Cu films was obviously unimpaired under such thermal bonding and a few tiny voids were detected in the bonding interface. We further increased the bonding temperature to 200 °C and bonded for 30 min. Figure 8 shows the cross-sectional FIB micrographs of the CMP nt-Cu films bonded at 200 °C for 30 min. The bonding interface showed no signs of voids under such a bonding condition.

**Figure 9:**
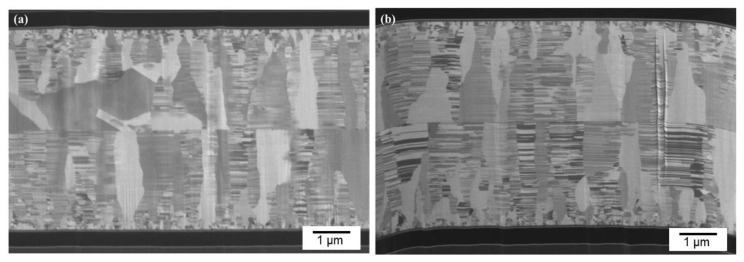
Cross-sectional FIB images (**a**,**b**) of the typical CMP nt-Cu films bonded at 200 °C for 10 min.

We further reduced bonding time using the same CMP nt-Cu films. Figure 9 shows the cross-sectional FIB images of the CMP nt-Cu films bonded at 200 °C for 10 min. We discovered that a few tiny voids are present in the bonding interface. Additionally, the bonding time was further reduced to 5 min. Figure 7b presents a cross-sectional FIB micrograph of the CMP nt-Cu films bonded at 200 °C for 5 min. A larger number of tiny voids existed in the bonding interface. We believe that such a Cu-to-Cu bonding was not as complete as samples under longer bonding time (30 min and 10 min).

## 4. Discussion

In order to understand the bonding mechanism of such nt-Cu films, the Arrhenius equation was employed to calculate the theoretical surface diffusivity of nt-Cu [36]. The diffusivities for (111), (100), and (110) planes are of 9.42 × 10^−6^ cm^2^/s, 1.19 × 10^−9^ cm^2^/s, and 5.98 × 10^−11^ cm^2^/s, respectively [39]. This indicates that the diffusion on the (111) surface is much faster than that on the other planes. We assume that the interfacial bonding of our nt-Cu films was achieved by a creep-induced deformation [12,23]. During the bonding process, high compressive stress was applied on the chips and nt-Cu films at an elevated temperature [40]. The contact regions were under severe compressive stress while the non-contact regions (voids) were subjected to less severe or no stress. This leads to a stress gradient where Cu atoms diffuse from the high localized to lower stress regions [41,42,43]. These Cu atoms slowly fill up voids and eventually form a bonding. Note that the nt-Cu films employed in this study possessed a high percentage of (111)-oriented surfaces (Figure 6). Thus, the direct Cu-to-Cu bonding was easily accomplished as a result of a fast diffusion of (111)-oriented nt-Cu surface [12,23].

**Figure 10:**
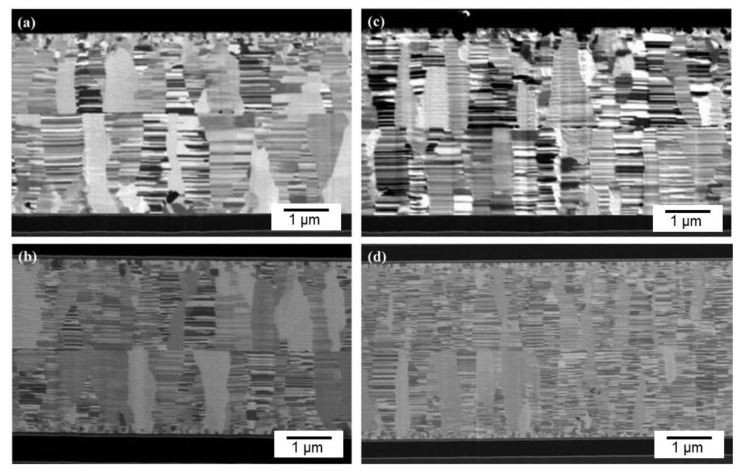
Bonding interfaces of the (**a**) electropolished nt-Cu films bonded at 150 °C for 60 min, (**b**) CMP nt-Cu bonded at 150 °C for 60 min, (**c**) electropolished nt-Cu bonded at 200 °C for 30 min, and (**d**) CMP nt-Cu bonded at 200 °C for 30 min.

**Figure 11:**
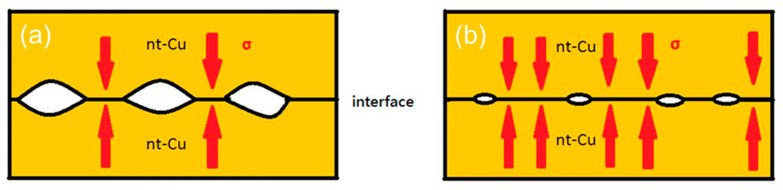
Schematic diagrams of pre-existing void scales at the bonding interfaces correlated with their surface roughness. (**a**) Large and (**b**) small void scales. (*σ* is denoted as the applied stress).

In order to investigate the effect of CMP treatment on bonding quality, some nt-Cu films were treated using a conventional electropolishing method and bonded for comparison. Figure 10 presents the typical bonding interfaces of the CMP and electropolished films. As shown in Figure 10, the CMP nt-Cu bonding exhibited a greater quality with a few tiny voids in the interface compared to that of the electropolished ones. As aforementioned, the CMP Cu-to-Cu bonding was accomplished in a shorter time due to the lower roughness (Figure 5). It can be ascribed to the smaller number and scale of pre-existing voids in the bonding interface. Figure 11 shows the schematic diagrams of pre-existing voids corresponding with the surface roughness scales. Note that the roughness of the electropolished nt-Cu films was greater than that of the CMP nt-Cu ones (Figure 5). Therefore, the scale of pre-existing voids was larger, as illustrated in Figure 11. Assuming a bonded sample with similar diffusivity and diffusion rate; it might take a longer time to fill up a larger void. Thus, the CMP nt-Cu films bonded with the same bonding time and temperature showed a greater quality (Figure 10). In addition, the bonding time of such flattened CMP nt-Cu films could be significantly shortened to 10 min and the columnar nt structure remained unchanged after the bonding procedure.

**Figure 12:**
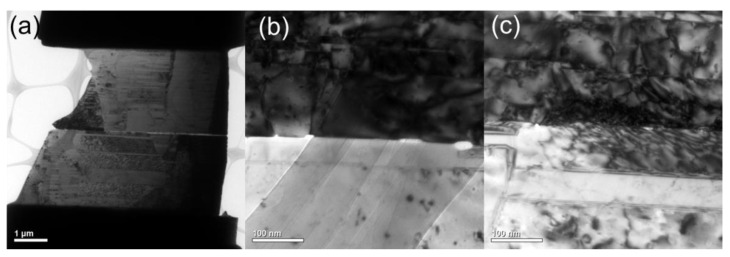
(**a**) Cross-sectional TEM image of the typical CMP nt-Cu films bonded at 200 °C for 10 min. (**b**,**c**) Enlarged images of (**a**).

**Figure 13:**
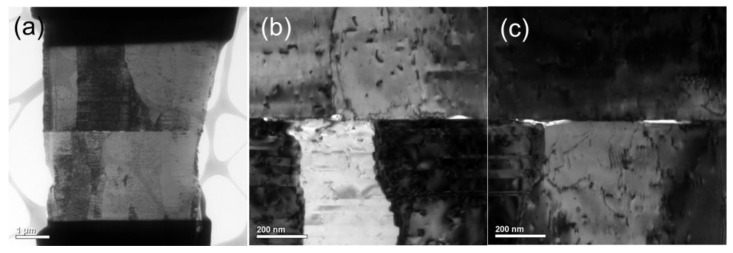
(**a**) Cross-sectional TEM image of the typical CMP nt-Cu films bonded at 200 °C for 5 min. (**b**,**c**) Enlarged images of (**a**).

In addition, for comparison, we further examined the bonding interfaces of the CMP nt-Cu films bonded at 200 °C for 10 min and 5 min using XTEM (Figure 12 and Figure 13). The typical interface of the Cu-to-Cu samples bonded for 10 min presented a few tiny voids (Figure 12b). Such voids were much smaller compared to that of the sample bonded for 5 min (Figure 13b,c). However, the void number that exists was almost identical and the bonding interfaces were extraordinarily smooth for all bonded samples. This indicates that such bonding conditions using our CMP nt-Cu films effectively suppressed residual voids in the interface and produced an excellent bonding quality. Table 1 lists the characteristics of Cu-Cu bonding using different surface treatments. Obviously, the CMP treatment can offer great bonding quality and low bonding temperature. The Cu-to Cu direct bonding conditions using the nt-Cu films are thus applicable for rapid 3D ICs integration and other electronics industries.

## 5. Conclusions

In summary, we employed cross-sectional and plan-view TEM imaging to quantitatively characterize the interfacial bonding quality of the Cu-to-Cu films. Highly (111)-oriented nt-Cu films polished using CMP and electropolishing were successfully bonded under various thermal conditions, ranging between 150 to 200 °C. Such low-temperature Cu-to-Cu direct bonding was virtually free of voids with a great bonded interface. The bonding process was accomplished by a fast diffusion of Cu atoms on the (111) surface. It was also attributed to the role of creep-induced deformation in eliminating pre-existing voids under such bonding processes. In addition, we discovered that the CMP nt-Cu bonding quality was greater than that of the electropolished nt-Cu films. This is due to the fact that the CMP nt-Cu film possessed a very low surface roughness. It thus resulted in the smaller scales of pre-existing voids and played a crucial role in Cu-to-Cu direct bonding. Owing to the low surface roughness of the CMP nt-Cu films, the bonding time could be further reduced to 10 min. Such nt-Cu shows great potential for low-temperature direct Cu-Cu bonding in ultra-fine pitch packaging. In addition to the nt-Cu application, surface passivation is a promising technique to reduce the thermal budget and enhance the bonding strength. This study is hoped to provide a favorable method in advancing the current 3D ICs packaging technology.

## Figures and Tables

**Table 1:** Summary of the recent Cu-Cu bonding technologies.

	Surface Treatment	Bonding Temp. (°C)	Bonding Strength	Surface Roughness (nm)	Duration (min)
This work	CMP	150–200	High	2.4	5–60
Tseng et al. [44]	Electropolishing	120–400	High	5.8	30–240
Liu et al. [12]	Chemical cleaning	200–250	High	N/a	10–60
Liu et al. [23]	Electropolishing	150–250	High	6.5	30–60
Huang et al. [45]	Electropolishing	300	High	22	30
Liu et al. [46]	Passivation	70–150	Low	2.5	15
Liu et al. [47]	Passivation	70–200	Low	5	15
Chang et al. [48]	Electropolishing	250–350	High	6.6	5–90
Wu et al. [49]	Passivation	200–300	Low	1.0	20

## Data Availability

The raw/processed data required to reproduce these findings cannot be shared at this time due to legal or ethical reasons.
